# Roxadustat regulates the cell cycle and inhibits proliferation of mesangial cells via the hypoxia-inducible factor-1α/P53/P21 pathway 

**DOI:** 10.3389/fcell.2025.1503477

**Published:** 2025-02-18

**Authors:** Yun Cheng, Qingmei Yang, Baijie Feng, Xiuhong Yang, Huimin Jin

**Affiliations:** ^1^ Division of Nephrology, Shanghai Pudong Hospital, Pudong Medical Center, Fudan University, Shanghai, China; ^2^ Nephrology Department, Shanghai Public Health Clinical Center, Fudan University, Shanghai, China; ^3^ Division of Oncology, Shanghai Pudong Hospital, Pudong Medical Center, Fudan University, Shanghai, China; ^4^ Department of Nephrology, Huadong Hospital, Fudan University, Shanghai, China; ^5^ Division of Hemodialysis, Shanghai Dong Ji Fresenius Hemodialysis Center, Shanghai, China

**Keywords:** diabetic nephropathy, mesangial cells, cell cycle, cell proliferation, HIF-1α

## Abstract

**Background:**

Over-proliferation of mesangial cells (MCs) is one of the main pathological changes in the early stages of diabetic kidney disease (DKD). Roxadustat, an oral hypoxia-inducible factor (HIF) prolyl hydroxylase inhibitor, is widely studied in different models of kidney disease. Whether roxadustat has beneficial effect on the proliferation of MCs and DKD remains unknown.

**Methods:**

The optimal concentration of roxadustat for inhibiting MC proliferation was determined using CCK-8 and colony formation assays. Changes in the cell cycle were detected using flow cytometry, and changes in cell cycle-related proteins were screened using quantitative reverse transcription polymerase chain reaction. Reverse experiments were applied using *Trp53-/-* cell line. Co-immunoprecipitation was used to verify the interaction between HIF-1α and p53 and predict sites of interaction. Finally, a corresponding *in vivo* verification was performed.

**Results:**

Optimal concentrations of high glucose (30 mM) and roxadustat (100 μM) were established. Roxadustat showed anti-proliferation effect on MCs through S-phase arrest. HIF-1α/p53/p21 and downstream cyclins (cyclin A1, cyclin A2, and cyclin E1) showed corresponding changes. A reverse experiment confirmed that HIF-1α affected cell cycle and proliferation through p53, and co-immunoprecipitation results showed a interaction between HIF-1α and p53. Molecular docking predicted the possible interaction between Lys328, Pro332, Arg245, and Lys251 of HIF-1α and Ala222, Tyr226, Glu225, and Asp265 of p53, respectively. Finally, animal experiments demonstrated similar effect of roxadustat in db/db mice.

**Conclusion:**

Roxadustat regulates and inhibits cell proliferation of MCs via the HIF-1α/p53/p21 pathway. This may be a potential therapeutic target in the early stages of diabetic kidney disease.

## Introduction

Diabetic kidney disease (DKD) is one of the major causes of end-stage renal disease, which has caused a huge social and economic burden (GBD Chronic Kidney Disease Collaboration, 2020; Zhang et al., 2020). It is characterized by mesangial cell (MCs) proliferation, massive accumulation of extracellular matrix, thickening of the basement membrane, and interstitial fibrosis (Tervaert et al., 2010). Over-proliferation of MCs and accumulation of mesangial matrix are the main pathological changes in the early stage of DKD, which gradually progress to membrane thickening and glomerulosclerosis (Avraham et al., 2021). Sustained high-glucose stimulation has been reported to lead to over-proliferation of MCs and accumulation of the extracellular matrix (Chen et al., 2019a). Several reports of different pathways exist, including Akt/mTOR, AMPK/SIRT, and PI3K/AKT/GSK-3β, which mediates autophagy, reactive oxygen species (ROS), and inflammatory response, respectively (Chen et al., 2021; Lei et al., 2023).

Roxadustat, an oral hypoxia-inducible factor prolyl hydroxylase inhibitor (HIF-PHI), is the first oral HIF-PHI drug on the market and has been widely used for the treatment of renal anemia whether on dialysis or not (Chen et al., 2019b; Chen et al., 2019c; Provenzano et al., 2021a; Provenzano et al., 2021b). Recent studies also found its potential abilities for the treatment of other diseases (Zhu et al., 2022). It inhibits HIF-prolyl hydroxylase (HIF-PHD) by mimicking its substrate to stabilize hypoxia-inducible factor-1α (HIF-1α), which is degraded under normoxic condition (Locatelli and Del Vecchio, 2022). In other words, roxadustat increases the concentration of HIF-1α under normal oxygen. HIF-1α is translocated into the nucleus and forms a dimer with HIF-1β to interact with hypoxia response elements (HRE).

Overactivation of HIF-1α is discovered in some renal disease models. However, there are controversies regarding the effect of elevation of HIF-1α. Wang’s study showed that overactivation of HIF-1α is a pathogenic factor in chronic renal injury induced by ischemia (Wang et al., 2014). While in the folic acid-induced kidney injury model, roxadustat pretreatment showed a protective role by decreasing ferroptosis at the early stage via upregulation of HIF-1α and Akt/GSK-3β-mediated Nrf2 activation, which retards the fibrosis progression subsequently (Li et al., 2020). Besides, another study suggested that roxadustat had little effect on renal fibrosis even though a high dose of roxadustat transiently (7 days) potentiated gene expression of profibrogenic molecules, including plasminogen activator inhibitor 1 (Pai-1) and connective tissue growth factor (Ctgf) in the UUO model (Kabei et al., 2020). The role of HIF-1α varies in disease models of non-diabetic nephropathy.

For DKD, studies are lacking. Recently, a study revealed that roxadustat improved DKD phenotypes by reconstructing the intestinal microbial profiles and upregulating the production of gut-associated beneficial metabolites (Jiang et al., 2023). Xie et al. suggested that roxadustat ameliorated high glucose-induced rat glomerular endothelial cells (rGECs) injury by upregulating HIF expression, indicating that FG-4592 might be favorable for further DN treatment (Xie et al., 2019). However, another study suggested that HIF-1α was involved in cell cycle regulation and MCs proliferation induced by high glucose (Cao et al., 2019). None of the above has been studied in depth.

Therefore, this study aimed to investigate whether roxadustat could inhibit the proliferation of MCs via upregulation of HIF-1α and its underlying mechanism in depth. These findings might be helpful for further study regarding the treatment of DKD.

## Materials and methods

### Chemicals

Roxadustat (FG-4592) (Selleck, United States) was purchased in powder form and dissolved in dimethyl sulfoxide (DMSO) (Beyotime, Cat no ST038, China) at a concentration of 200 mM for *in vitro* experiments.

Roxadustat was prepared as a 10 mg/mL solution in the vehicle.10 mg roxadustat was dissolved in 50 μL DMSO, 400 μL liquid PEG 300 (Selleck, Cat no. sS6704, United States), 50 μL Tween 80 (Selleck, Cat no. S6702, United States) and 500 μL ddH2O in sequence. Mice were treated with 10 mg/kg FG-4592 or vehicle alone by oral gavage daily.

### Stable cell line construction

The SV40 MES 13 cell (mouse MCs) line was obtained from the Chinese National Collection of Authenticated Cell Cultures (Shanghai, China). The MCs were cultured in the DMEM medium of 5.5 mM glucose supplemented with 5% fetal bovine serum (FBS) (Invitrogen) at 37°C in a 5% CO_2_ atmosphere.

The sgRNAs of MCs were synthesized by Genomeditech, Inc (Shanghai, China) and cloned into the lentiCRISPRv2 lentiviral vector to construct the lentiCRISPR-p53 knockout plasmids (Table 1). The scrambled vector or target plasmid (p53 knockout plasmids), psPAX2, and PMD.2G were co-transfected into HEK293T cells to synthesize a scrambled control lentivirus or p53 knockout lentivirus according to the instruction of Lipofectamine 3,000 (Invitrogen, United States). When MCs reached 50% confluency, the cells were infected with the lentivirus and screened with puromycin treatment. The p53 knockout cell lines, MCs-KO1, MCs-KO2 and KONC (negative control for knockout), were screened using puromycin. Finally, the effect of the knockout was confirmed using Western blotting.

**TABLE 1:** sgRNA sequences of *Trp53-*KO1.

Name		Primers for PCR (5′–3′)
*Trp53-*KO1	Forward	CAC​CGT​CGT​CCA​TGC​AGT​GAG​GTG​A
	Reverse	AAA​CTC​ACC​TCA​CTG​CAT​GGA​CGA​C
*Trp53-*KO2	Forward	CAC​CGA​ACA​GAT​CGT​CCA​TGC​AGT​G
	Reverse	AAA​CCA​CTG​CAT​GGA​CGA​TCT​GTT​C

### Cell culture and treatment

After MCs adhered to the culture dishes (about 12 h), the medium was exchanged with low-glucose DMEM (5.5 mM) without FBS for 24 h to synchronize the cell cycle. After 24 h, the cells were divided into the following groups:

1) The control (Ctrl) group (cultured in DMEM medium containing 5.5 mmol/L glucose with 1% FBS).
2) The isotonic (MA) group (cultured in DMEM medium containing 5.5 mmol/L glucose +24.5 mmol/L mannitol with 1% FBS) (This group was included to eliminate the effects of osmotic pressure).
3) The high-glucose (HG) group [cultured in DMEM medium containing 30 mmol/L glucose (no mannitol) with 1% FBS].
4) The roxadustat (HG + Rot) group [cultured in DMEM medium containing 30 mmol/L glucose (no mannitol) and 10–200 μmol/L roxadustat with 1% FBS].

The cells were collected for assessments at 24 h, 48 h, 72 h.

### Cell counting kit-8

In this study, 3 × 10^3^ cells were seeded in 96-well plates and cultured as described above. 10 μL of cell-counting kit-8 (CCK-8) (Dojindo Molecular Technologies, Cat no. ck04, Japan) solution was added to each well and incubated for 2 h. The absorbance of each well was measured at a 450 nm wavelength.

### Colony formation

In this study, 500 cells were seeded in six-well plates and cultured at 37°C. The assay was terminated when the majority of clones formed (>50 cells per colony). After observation, the clones were fixed with 4% paraformaldehyde for 30 min at room temperature. Subsequently, the plates were stained with a 0.5% crystal violet solution for 30 min at room temperature and washed three times (5 min per time) with phosphate-buffered saline. The cells were observed under a 4 × light microscope (Yuyan Instruments, Shanghai, China) and photographed. Clone formation rate (%) = (number of clones/500) × 100.

### Flow cytometric analysis of cell cycle distribution

A total of 1 × 10^6^ cells were collected by centrifugation (1,000 rpm, 5 min) at 25°C, and fixed in 70% ethanol, and then the cells were incubated at −20°C for 24 h. Subsequently, the samples were stained with propidium iodide (PI) staining solution for 30 min at 25°C protected from light and analyzed using flow cytometry (BD Biosciences, United States). The DNA content in the cells was analyzed according to the fluorescence intensity, and the percentage of the number of cells in G1/G0, S, and G2/M phases is determined. Percentages of different cell cycle were calculated using Modfit software (Verity Software House, United States).

### RNA extraction and quantitative RT-qPCR

Total RNAs were extracted from each group using TRIzol reagent (Invitrogen Life Technologies, United States). Complementary DNA (cDNA) was synthesized using a PrimerScript real-time reagent kit (Takara Biotechnology, China). The ABI 7900HT RT-qPCR system (Applied Biosystems Life Technologies, CA, United States) was used in triplicates. The primers used are shown in Table 2. Comparative cycle threshold values (2^−ΔΔCT^) were applied to analyze the final results.

**TABLE 2:** Sequences of primers for RT-qPCR.

Name		Primers for PCR (5′–3′)
*Trp53*	Forward	CCC​CTG​TCA​TCT​TTT​GTC​CCT
	Reverse	AGC​TGG​CAG​AAT​AGC​TTA​TTG​AG
*Cdkn1a*	Forward	CCT​GGT​GAT​GTC​CGA​CCT​G
	Reverse	CCA​TGA​GCG​CAT​CGC​AAT​C
*Ccna1*	Forward	ACA​TGG​ATG​AAC​TAG​AGC​AGG​G
	Reverse	GAG​TGT​GCC​GGT​GTC​TAC​TT
*Ccna2*	Forward	AAG​AGA​ATG​TCA​ACC​CCG​AAA​AA
	Reverse	ACC​CGT​CGA​GTC​TTG​AGC​TT
*Ccne1*	Forward	GTG​GCT​CCG​ACC​TTT​CAG​TC
	Reverse	CAC​AGT​CTT​GTC​AAT​CTT​GGC​A
*Actb*	Forward	GGC​TGT​ATT​CCC​CTC​CAT​CG
	Reverse	CCA​GTT​GGT​AAC​AAT​GCC​ATG​T

### Mice model and treatment

This study adhered to ARRIVE (Animal Research: Reporting of *In Vivo* Experiments) guidelines to ensure transparent and comprehensive reporting of all animal experiments. All animal experiments were approved by the Institutional Animal Care and Utilization Committee of the Animal Experimentation Center of Pudong, Fudan University (Grant No. 2020-MS-Zdxk-10).

Male C57BLKS/J db/db and db/m mice (males, 8 weeks) were purchased from GemPharmatech (Nanjing, China). All the mice were housed in plastic cages within the following environment: the temperature was 23°C–26°C, the humidity was 50%–60%, and a 12 h light/dark cycle. All the mice were fed high-fat and high-protein food for 12 weeks till harvest.

The mice were divided into three groups: db/m (N = 10, treated with the corresponding solvent), db/db (N = 10, treated with the corresponding solvent), and db/db + Rot (N = 10, treated with roxadustat solvent). Roxadustat was administered by gavage at a concentration of 30 mg/kg per day for 12 weeks. End weight, kidney index, hemoglobin, fasting blood glucose, serum creatinine, and 24 h urine protein levels were measured during harvest.

### Measurement of biochemical indicators

Levels of hemoglobin, fasting blood glucose, serum creatinine, and urine protein were determined using assay kits, according to the manufacturer’s instructions (Nanjing Jiancheng Bioengineering Institute, China). Each mouse was placed in a separate metabolic cage and had free access to food and water. The urine of mice was collected between 8 a.m. and 8 a.m. the next day. A 24 h urine protein = 24 h urine volume × urine protein concentration.

### Hematoxylin & Eosin staining and Masson’s staining

At termination, the kidneys were weighed, fixed with 4% paraformaldehyde, embedded in paraffin, and cut into 4 μm thick sections. Subsequently, H&E staining, Masson’s staining and were performed.

### Periodic acid-schiff staining and glomerular volume

The kidneys were fixed in 4% paraformaldehyde and embedded in paraffin. Then, the kidneys were sliced into transverse sections (4 μm) for PAS staining (Periodic acid oxidation, Scheff’s reagent staining, and hematoxylin staining were sequentially performed). The PAS staining morphology of the kidney tissues was observed under a light microscope.

$$\text{Glomerular Volume}={\left(A\right)}^{3/2}\times \beta /d$$

Where A is the mean glomerular area and was measured as reported (Lopes et al., 2005), β is 1.38, which is the shape coefficient of the sphere, and d is 1.01, which is the size distribution of glomeruli, assuming a 10% coefficient of variation of the caliber diameter.

### Immunohistochemistry

Kidneys were fixed in paraformaldehyde for immunohistochemistry (IHC) and were paraffin-embedded and sectioned. The paraffin section was deparaffinized and rehydrated. The microwave method was used for later antigen retrieval. Slides were incubated overnight at 4°C in the blocking solution containing primary antibodies, followed by incubation with biotin-labeled secondary antibodies and streptavidin-horseradish peroxidase complex (Beyotime, Cat no. P0615, China). Afterward, diaminobenzidine staining and hematoxylin counterstaining were applied. Primary antibodies were as follows: PCNA (Cell Signaling, Cat no. 13110s, 1:100), Ki-67 (Abcam, Cat no. ab15580, 1:100), HIF-1α (Proteintech, Cat no. 20960-1-AP, 1:500), p53 (Proteintech, Cat no. 21891-1-AP, 1:200), p21 (Cell Signaling; Cat no. 2947s, 1:200).

### 5-Ethynyl-2′-deoxyuridine assay

The mice were administered intraperitoneal injections of 5 mg/kg EdU solution daily for 2 weeks before harvest. The mice were then sacrificed, and their kidneys were separated and processed for EdU detection according to the manufacturer’s protocol (Click-IT Assay Kit, Invitrogen, United States).

### Western blotting

Total cellular proteins from each group were extracted using radioimmunoprecipitation assay lysis buffer containing protease and phosphatase inhibitors. Total protein concentrations were determined and regulated using a BCA protein assay kit (Thermo Fisher Scientific, United States). The same volume (10–20 μg) of each group was loaded per lane. After the proteins were separated on 6%–10% SDS-PAGE gels, they were transferred to 0.45 μm polyvinylidene difluoride membranes (Millipore, United Kingdom) at 400 mA for 45 min. The membranes were then blocked with 5% non-fat milk for 2 h at room temperature and incubated with primary antibodies overnight at 4 C. Secondary antibodies were anti-mouse or anti-rabbit antibodies and were conjugated to horseradish peroxidase (Abclonal, China, 1:5000), and incubated at room temperature for approximately 2 h. Finally, the bands were visualized with enhanced chemiluminescence reagents (Thermo Fisher Scientific, United States) and developed by Omega Lum G (Aplegen, United States). The Western blot bands were quantified using ImageJ Software (version 1.41). The following antibodies were tested: HIF-1α (Proteintech, Cat no. 20960-1-AP, 1:1000), p53 (Proteintech, Cat no. 21891-1-AP, 1:1000), p21 (Cell Signaling; Cat no. 2947s, 1:1000), cyclin A1 (Abclonal, A14529, 1:1000), cyclin A2 (Abclonal, A19036, 1:1000), cyclin E1 (Abclonal, A14225, 1:1000), and β-actin (Abclonal, AC026, 1:1000).

### Co-immunoprecipitation

The total cellular proteins (1 mg) of each group were extracted using NP-40 lysis buffer, and the primary antibody HIF-1α (Proteintech, Cat no. 20960-1-AP, 2 μg for 1 mg of total protein) was added to form a protein-antibody complex overnight at 4°C. The pre-washed magnetic beads (Thermo Scientific, Cat no. 88803, United States) were incubated with the protein-antibody complex for 1 h at room temperature. Then, the protein-antibody-magnetic bead complexes were collected, the magnetic beads, proteins, and antibodies were separated, and Western blotting was performed. The following antibody was tested: p53 (Proteintech, Cat no. 21891-1-AP, 1:1000).

### Molecular docking verification

The amino acid sequence and crystal structures of the proteins were queried using the UniProt database (https://www.uniprot.org/). The molecular structural formula of the p53 protein (PDB: 3EXL) was obtained from the RCSB PDB database (https://www.rcsb.org/). The structure of Hif-1α was obtained from Uniprot using an alphafold-generated three-dimensional structure. All the docking calculations were performed using the Rosetta docking method. First, the protein structures were preprocessed using the Rosetta docking_prepack_protocol.static.linuxgccrelease structure preparation module. During the docking process, the main chain was fixed, and the protein-protein docking was performed using a global algorithm (docking_protocol.static.linuxgccrelease). Fifty structures were generated at each starting point. The lower the binding energy, the better the docking results. The Pymol 2.5.2 software was used to analyze the docking results.

### Statistical analysis

All data are shown as mean ± standard deviation. Statistical differences were analyzed and visualized using GraphPad Prism 8 software. One-way analysis of variance was employed to compare differences among groups, and Tukey’s test was performed to show differences between two groups. Statistical significance was considered at *P* < 0.05.

## Results

### Roxadustat inhibited high glucose-induced proliferation of MCs

**FIGURE 1:**
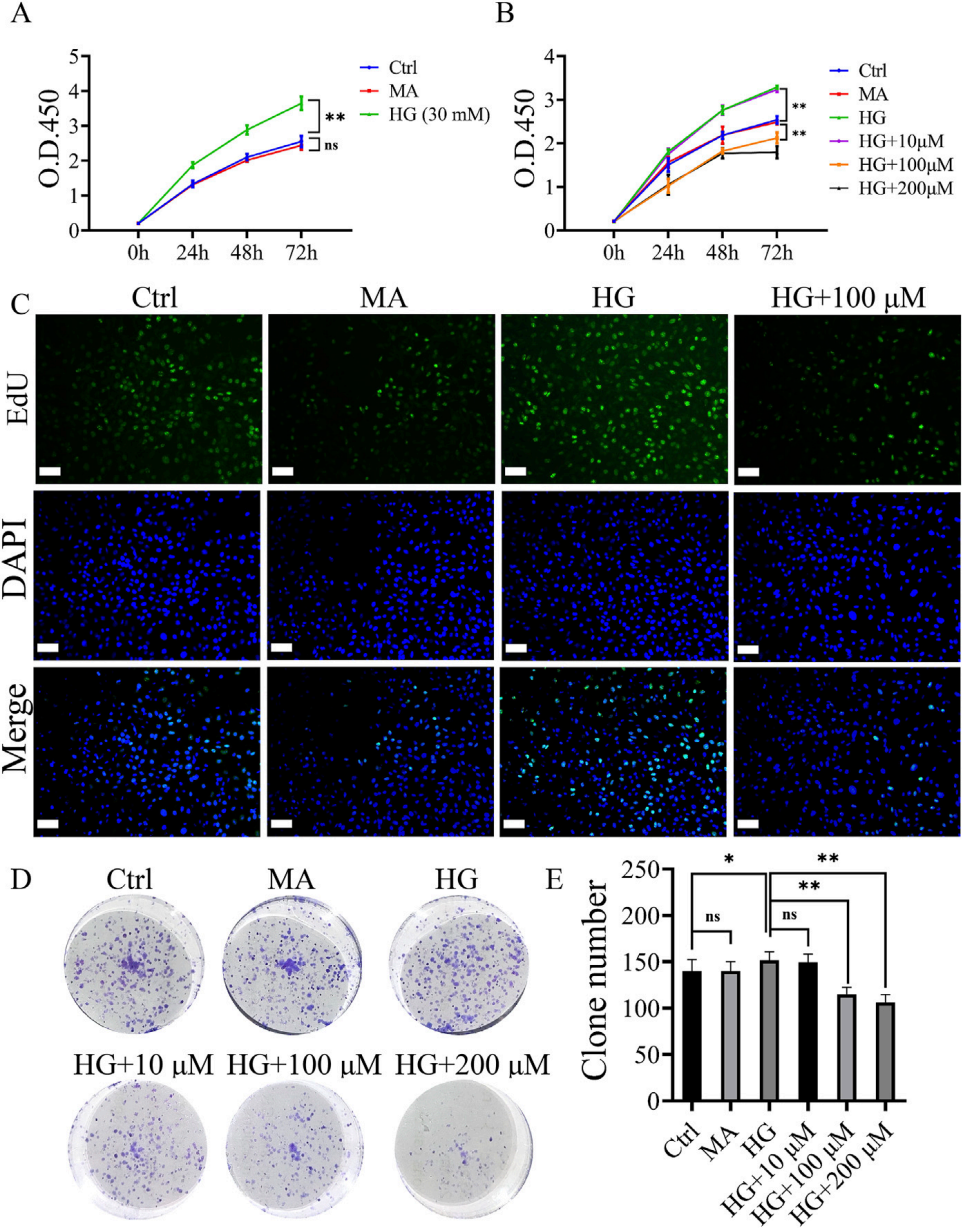
Roxadustat inhibited high glucose-induced proliferation of MCs. **(A)** O.D. 450 absorbance values at different concentrations of high glucose (Ctrl: 5.5 mM glucose, MA: 5.5 mM glucose + 24.5 mM mannitol, HG: 30 mM glucose). **(B)** O.D. 450 absorbance values at different concentrations of roxadustat (Ctrl: 5.5 mM glucose, MA: 5.5 mM glucose +24.5 mM mannitol, HG: 30 mM glucose, and HG + 10/100/200 μM: 30 mM glucose + 10/100/200 μM Roxadustat). **(C)** EdU pictures of different groups (scale bar: 50 μm). **(D, E)** Colony formation figures of different groups (ns: no significance, *P < 0.05, **P < 0.01).

Refer to Figure 1A. Compared with the MA groups, the viability of MCs in the Ctrl group showed no statistically significant. High glucose significantly promoted the proliferation of MCs. 30 mM was confirmed as the optimal concentration for this assay, as in most previous studies (Cao et al., 2019; Chen et al., 2019a).

The inhibitory effect of roxadustat on the proliferation of MCs gradually increased with increasing concentrations (10–200 μM), and the effect was most obvious at 72 h (*P* < 0.01). Based on the results of the colony formation experiment and CCK-8, a final concentration of 100 μM and duration of 72 h were selected for the subsequent experiments (Figures 1B–E).

### Roxadustat caused S-phase arrest in MCs via the HIF-1α/p53/p21 signaling pathways

**FIGURE 2:**
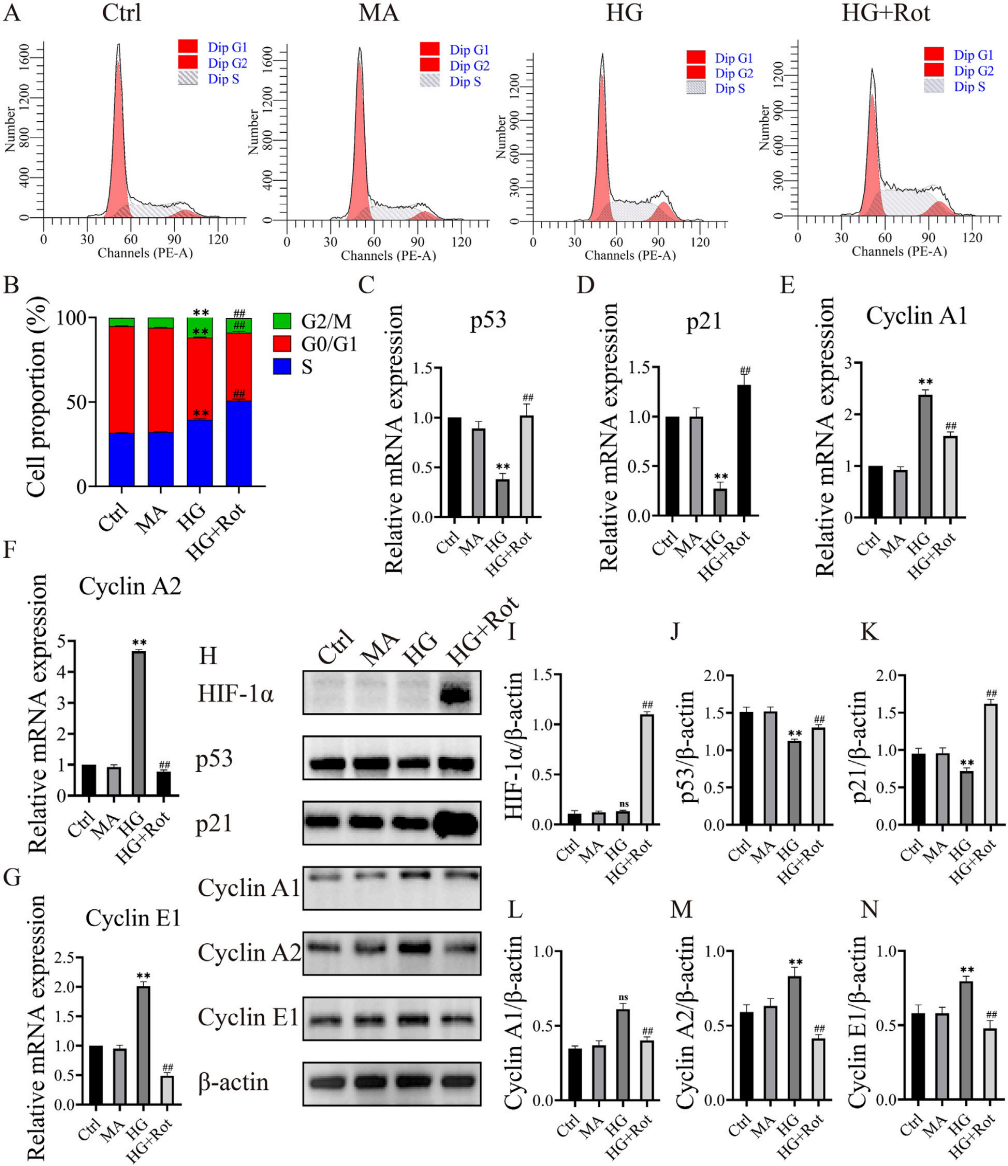
Roxadustat caused S-phase arrest in MCs via the HIF-1α/p53/p21 signaling pathways. **(A, B)** Cell cycle distribution in different groups. **(C–G)** Relative changes in the expression of different mRNA. **(H–N)** Changes in the expression of different proteins. (Ctrl: 5.5 mM glucose, MA: 5.5 Mm glucose +24.5 mM mannitol, HG: 30 mM glucose, HG + Rot: 30 mM glucose + 100 μM Roxadustat) (ns: no significance, **P < 0.01 HG vs. Ctrl, ^##^ *P* < 0.01 HG + Rot vs. HG).

Flow cytometry was applied to examine the cell cycle. Compared with the proportion of the HG group, the proportion of S-phase cells in the HG + Rot group was significantly higher (50.81% vs. 39.54%) (Figures 2A, B).

Given the changes in cell cycle, we subsequently screened the mRNA expression of cell cycle-related proteins using RT-qPCR. The relative mRNA expression of cyclin-dependent kinase inhibitors (*CDKIs*) (*Trp53* and Cdkn1a) in the HG group was significantly lower than that of the Ctrl and MA groups, and the trend reversed in the HG + Rot group (*P* < 0.01). The changing trend of *Cyclins* (Ccna1 *, Ccna2* and Ccne1) mRNA was opposite to that of *CDKIs* (*P* < 0.01) (Figures 2C–G).

Protein changes in p21, p53, cyclin A1, cyclin A2, and cyclin E1 were consistent with the mRNA changes (*P* < 0.01). Additionally, the HIF-1α level of the HG + Rot group was significantly higher than that of other groups. We assumed that roxadustat might regulate the proliferation and cell cycle of MCs through the HIF-1α/p53/p21 signaling pathways (Figures 2H–N).

### Knockout of Trp53 alleviated the anti-proliferative effect of roxadustat

**FIGURE 3:**
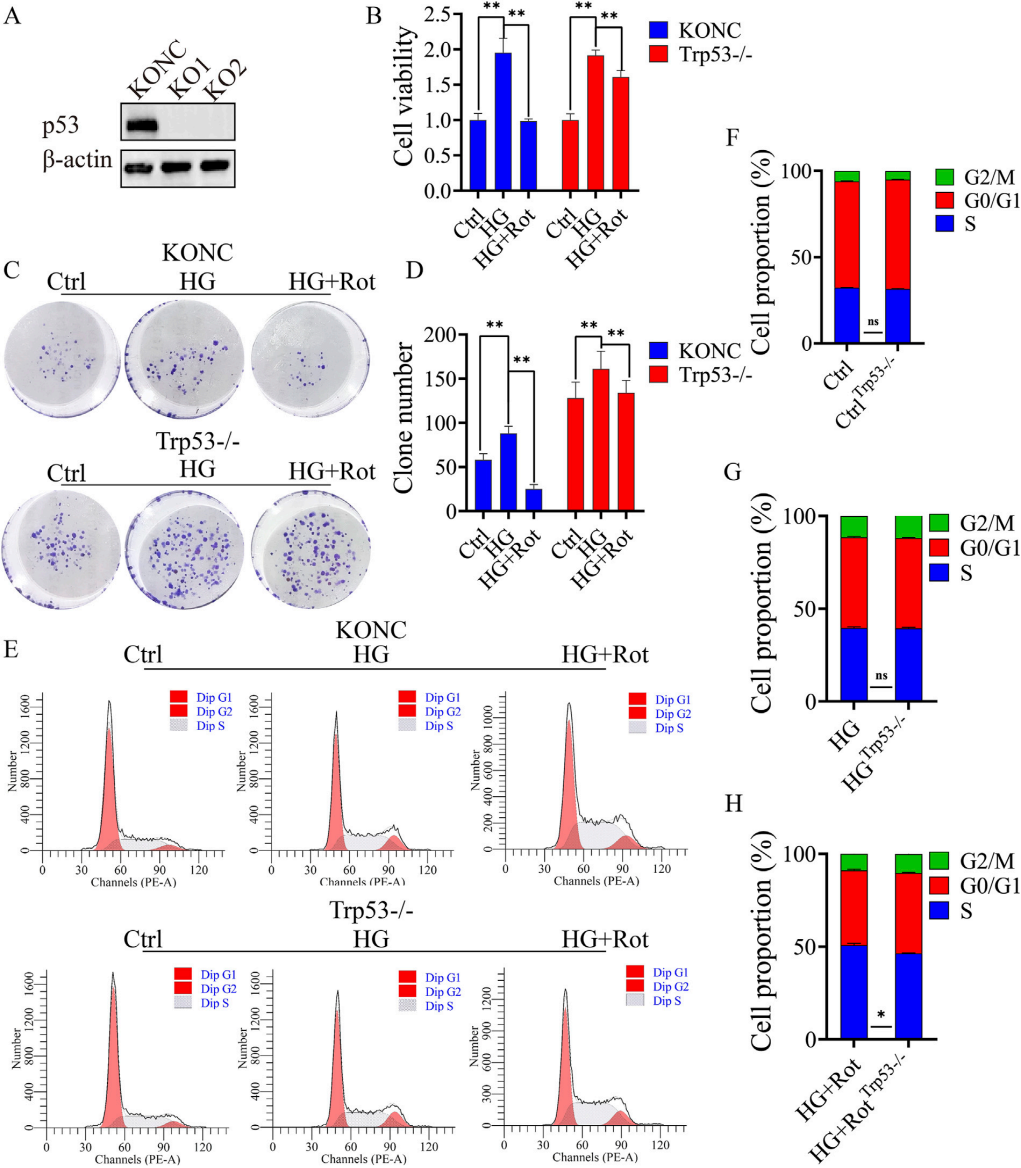
Knockout of Trp53 alleviated the proliferative inhibitory effect introduced by Roxadustat. **(A)** Verify Trp53 knockout effect in MCs by Western blot. **(B)** CCK-8 results of different groups in different cell lines. **(C, D)** Reverse studies of clone formation. **(E–H)** Cell cycle distribution in KONC and Trp53-/- cell lines (Ctrl: 5.5 mM glucose, MA: 5.5 mM glucose + 24.5 mM mannitol, HG: 30 mM glucose, HG + Rot: 30 mM glucose + 100 μM roxadustat, ns: no significance, **P < 0.01).

Two Trp53 knockout cell lines of MCs, KO1 and KO2, were established and confirmed (Figure 3A). The KO2 cell line was selected for subsequent experiments. Compared with the Ctrl group, the MA group displayed no significant differences in terms of proliferation, percentage of apoptosis, distribution of cell cycle, and expression of related proteins. Therefore, we designed three groups instead of four for further exploration: Ctrl, HG, and HG + Rot groups.

Follow-up experiments confirmed that the proliferation and S-phase arrest of the HG + Rot group in the Trp53-/- cell line were reversed when compared with those in the KONC cell line (Figures 3B–H).

In summary, Trp53 knockout alleviated the inhibitory effect of roxadustat on cell proliferation.

### Roxadustat exerted anti-proliferative effect via the HIF-1α/p53/p21 pathway and molecular docking result of HIF-1α and p53 proteins

The KONC and Trp53-/- MCs cell lines were used to explore the mechanism of roxadustat’s anti-proliferative effect.

**FIGURE 4:**
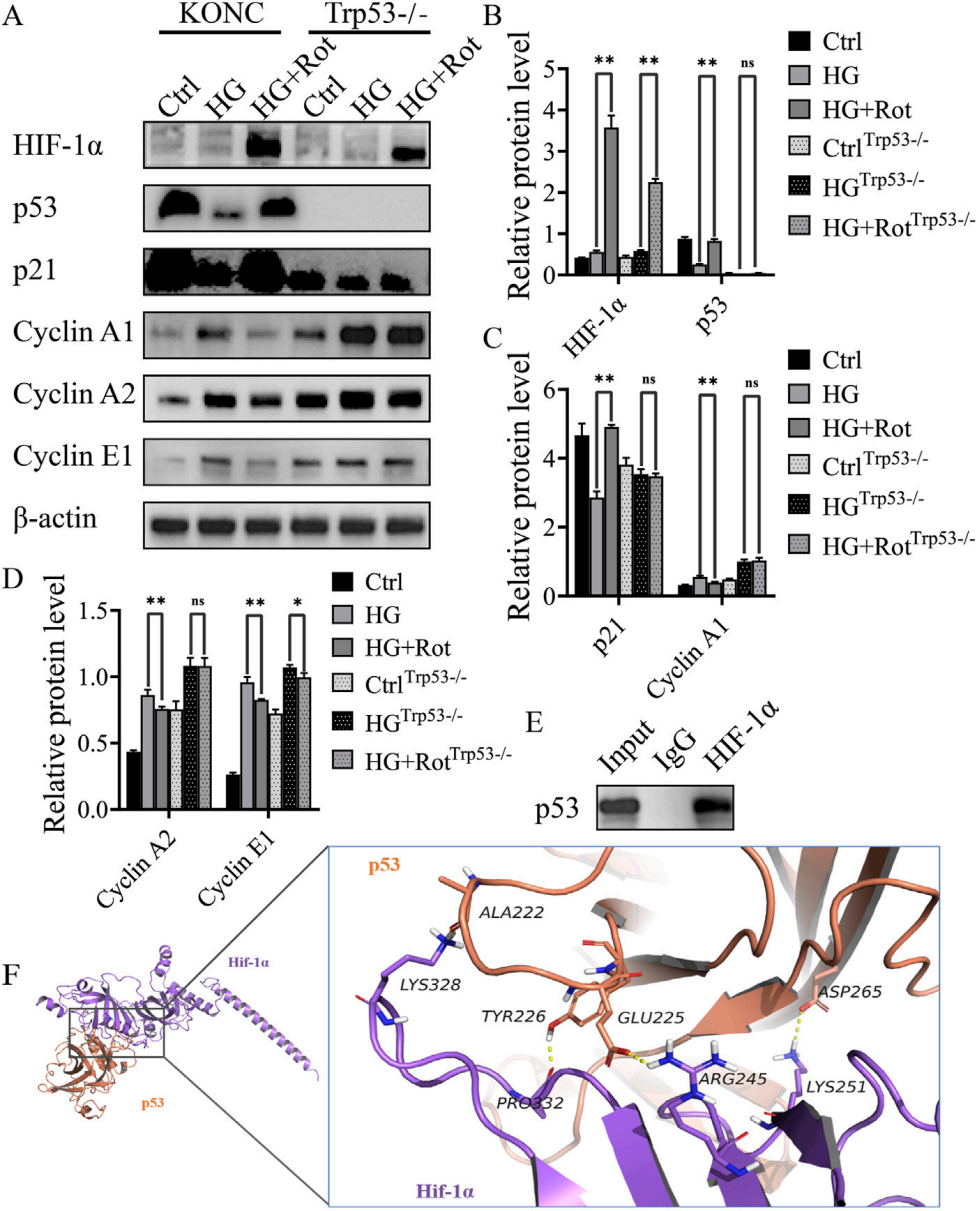
Roxadustat exerted anti-proliferative effect via the HIF-1α/p53/p21 pathway *in vitro* **(A–D)** Changes in the expression of different proteins. **(E)** CO-IP results. **(F)** Visual analysis of molecular docking of HIF-1α and predicted binding sites (Ctrl: 5.5 mM glucose, MA: 5.5 mM glucose + 24.5 mM mannitol, HG:30 mM glucose, HG + Rot: 30 mM glucose +100 μM roxadustat) (ns: no significance, *P < 0.05; **P < 0.01).

Western blot results showed that roxadustat significantly increased the expression of HIF-1α in both KONC and Trp53−/− cell lines. Compared with the KONC cell line, the overall expression of p53 disappeared, and p21 decreased, and its downstream cyclins (cyclin A1, cyclin A2, and cyclin E1) increased in the Trp53−/−cell line. The differences of expression of p21, cyclin A1, and cyclin A2 between the HG group and the HG + Rot group disappeared in the Trp53−/− cell line, and the difference of cyclin E1 decreased. This indicates that roxadustat exerted anti-proliferative effect via the HIF-1α/p53/p21 pathway and its downstream cyclins (Figures 4A–D).

Co-immunoprecipitation revealed a protein-protein interaction between HIF-1α and p53. Molecular docking predicted that Lys328, Pro332, Arg245, and Lys251 of HIF-1α might form a hydrogen bond with Ala222, Tyr226, Glu225 and Asp265 of p53, respectively. The docking score was −28.063 kcal/mol, which was much lower than the assessment criterion of −5 kcal/mol. The above results indicated that the four interaction sites might be the binding sites between HIF-1α and p53 (Figures 4E, F).

### Roxadustat improved renal function and relieved renal pathological lesion of db/db mice

**FIGURE 5:**
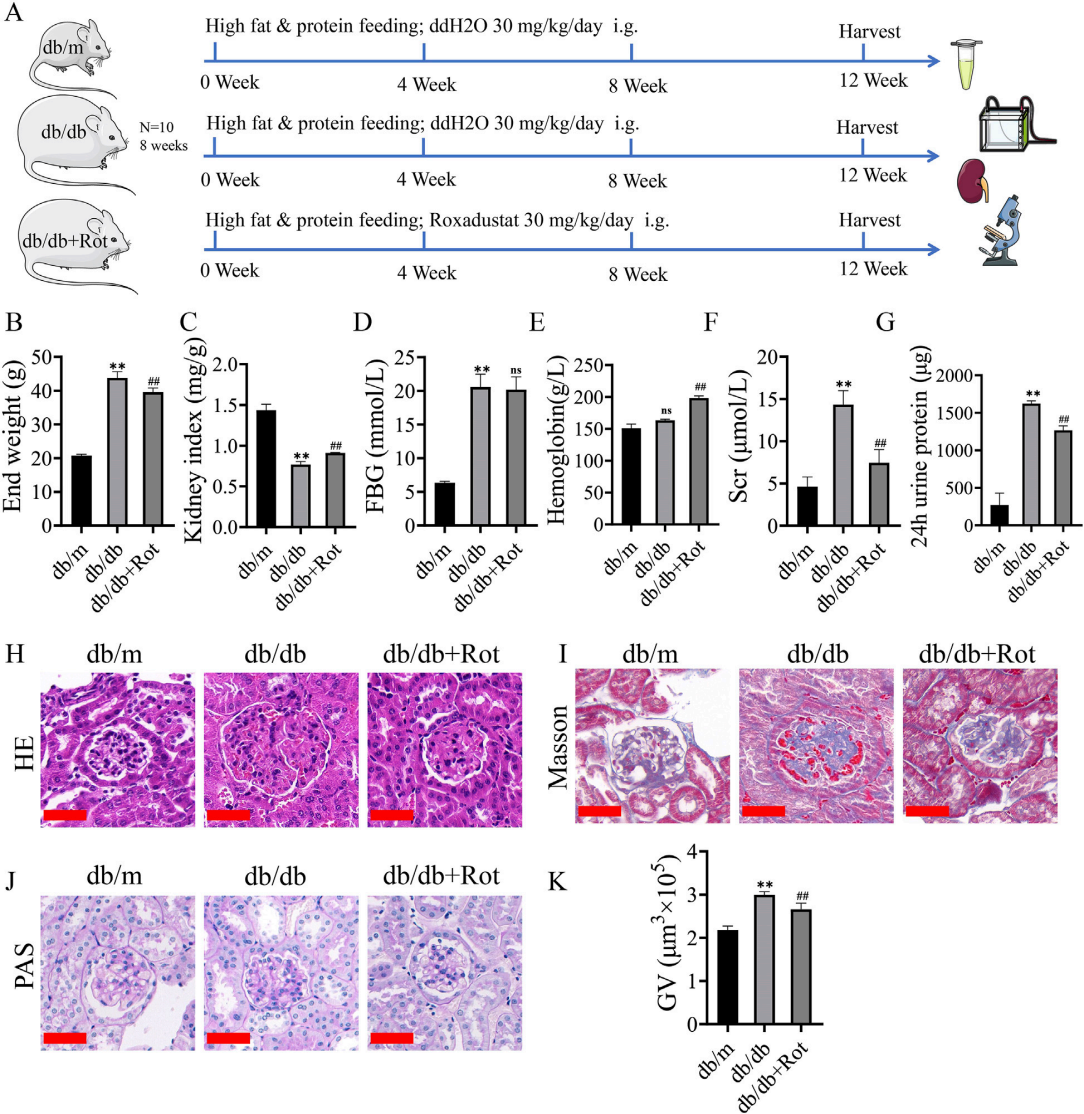
Roxadustat improved renal function and relieved renal pathological lesion of db/db mice. **(A)** Flow chart of *in vitro* experiments. **(B–G)** general condition and blood biochemical index results. **(H, I)** HE and Masson staining (scale bar: 50 μm). **(J, K)** PAS staining and glomerular volumes (scale bar: 50 μm). (db/m: heterozygous littermates, db/db: spontaneously diabetic mice, db/db + Rot: spontaneously diabetic mice + roxadustat treatment.) (^ns^: no significance, **P < 0.01 db/db vs. db/m, ^##^ *P* < 0.01 db/db + Rot vs. db/db).

The general condition and blood biochemical indices (end weight, kidney index, hemoglobin, fasting blood glucose, serum creatinine, and 24 h urine protein) of the db/db group showed corresponding changes of diabetes and DKD, and the db/db + Rot group showed varying degrees of improvement, except for fasting blood glucose (Figures 5A–G).

H&E showed glomerular hypertrophy and mesangial expansion, which were the features of DKD in the early stage, were reduced after the application of roxadustat (Figure 5H). Masson’s staining showed deposition of collagen fibers alleviated in HG + Rot group in mesangial areas of kidneys of db/db mice (Figure 5I). PAS staining showed that glomerular volume significantly decreased after the application of roxadustat (Figures 5J, K).

### Roxadustat exerted anti-proliferative effect via the HIF-1α/p53/p21 pathway in db/db mice

**FIGURE 6:**
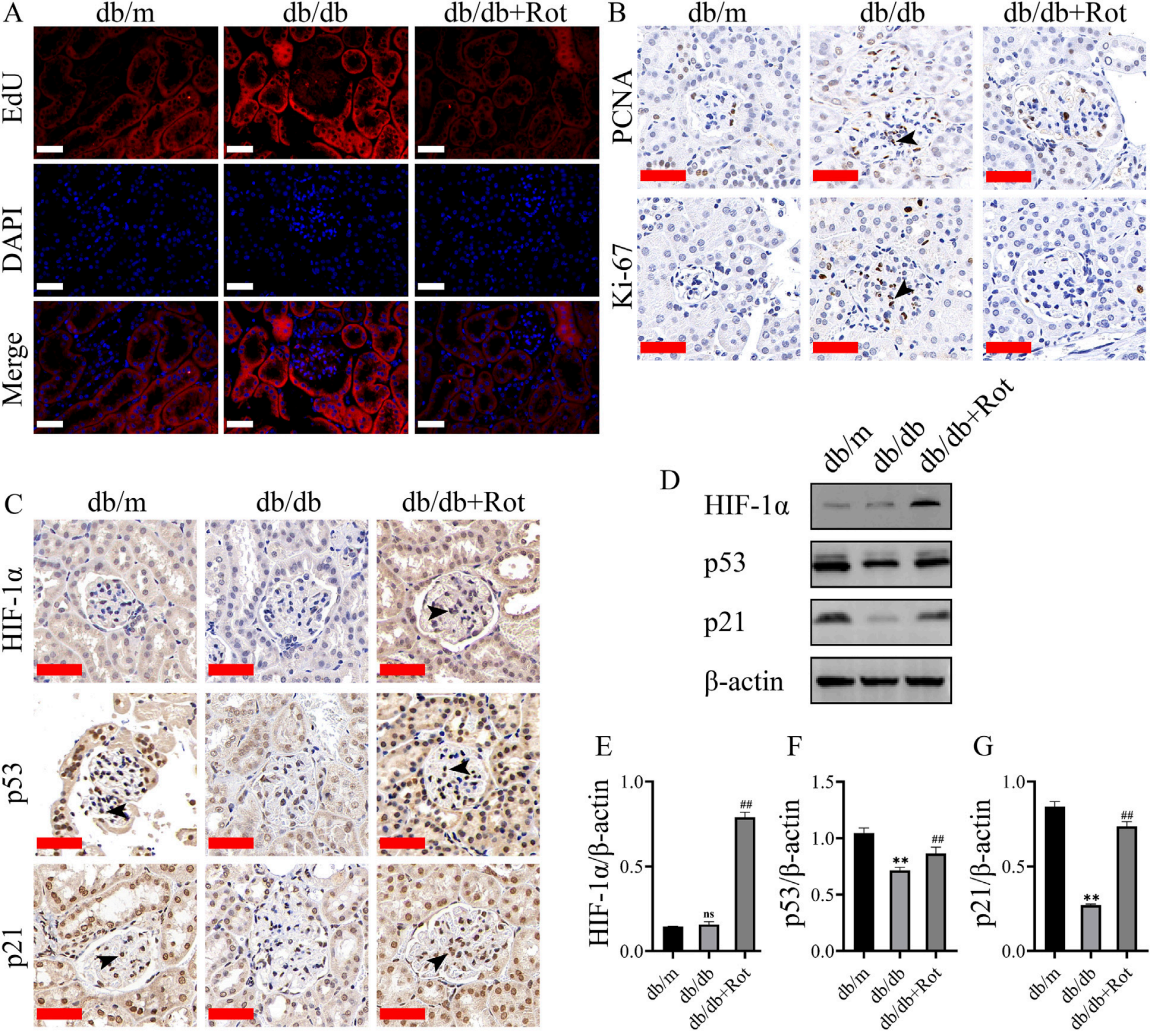
Roxadustat exerted anti-proliferative effect via the HIF-1α/p53/p21 pathway in db/db mice. **(A, B)** EdU, Ki-67, PCNA results result (scale bar: 50 μm, black triangle: positive staining). **(C–G)** Changes in the expression of different proteins (scale bar: 50 μm, black triangle: positive staining). (db/m: heterozygous littermates, db/db: spontaneously diabetic mice, db/db + Rot: spontaneously diabetic mice + roxadustat treatment.) (n^s^: no significance, **P < 0.01 db/db vs. db/m, ^##^ *P* < 0.01 db/db + Rot vs. db/db).

EdU, Ki67, and PCNA displayed over-proliferation of MCs in the db/db group than in the db/m group, and the proliferation of MCs was inhibited in the db/db + Rot group (Figures 6A, B).

Immunohistochemistry and Western blot results showed that roxadustat might inhibited the proliferation of MCs via the HIF-1α/p53/p21 pathway *in vivo*, which was consistent with the *in vitro* findings (Figures 6C–G).

## Discussion

The elevation of HIF-1α, a transcription factor, can improve the expression of its downstream HRE, which regulates the adaptation to hypoxia and reduces the harmful effect of hypoxia (Cabaj et al., 2022; Parveen et al., 2022). Numerous evidences suggest that roxadustat displayed a reno-protective ability in acute ischemic injury, post-renal transplantation patients, and new peritoneal dialysis (Wu et al., 2022; Yang et al., 2023). However, studies on the effect of roxadustat in DKD, both *in vivo* and *in vitro*, are lacking. In the present study, we confirmed that roxadustat had a reno-protective effect in db/db mice and inhibited the proliferation of mesangial cells induced by high glucose. Furthermore, we demonstrated that the reno-protective effect on DKD was exerted via HIF-1α/p53/p21 pathway.

Roxadustat increases the concentration of HIF-1α by reducing its degradation (Semenza, 2001; Kaelin, 2022). In some reports, inhibition of HIF-1α pathway reduced the high glucose-induced proliferation of glomerular MCs, while another HIF-PHI (FG-2216) reduced the proliferation of MCs in obese ZSF1 rats by increasing HIF-1α (Li et al., 2018; Signore et al., 2021). In addition, the dosage of roxadustat and experimental conditions varied both *in vivo* and *in vitro*. Pretreatment of roxadustat (10 mg/kg/day *in vivo* and 10 μM *in vitro*) protects against ischemia-induced acute kidney injury (AKI) via improving CD73 and decreasing AIM2 inflammasome activation (Yang et al., 2023). Administering roxadustat 50 mg/kg/day intragastrically for 14 days in streptozotocin (STZ) induced DKD mice reconstructed the intestinal microbial profiles of DKD (Jiang et al., 2023). There were no universal concentrations for the DKD model; the researchers took the intermediate concentration value, 30 mg/kg/da, as the action concentration in this study.

In this study, roxadustat reduced high glucose-induced MCs proliferation via cell cycle arrest and the change of mRNAs, such as *Trp53,* Cdkn1a, and downstream C*yclins* (*Ccna1*, Ccna2, and Ccne1). It is widely reported that p21 regulates Cyclin A1 (S and G2 phases), Cyclin A2 (S and G2 phases), and Cyclin E1 (late G1 and S phases). This might explain the S-phase stage induced by p53/p21 and was consistent with Chou’s report, which indicated the accumulation of p53 and p21 induced S-phase arrest in human renal tubular epithelial cells (Chou et al., 2022). However, this result is contrary to some articles which reported G1 arrest induced by the p53/p21/Rb pathway (Engeland, 2022). In our study, the RT-qPCR results did not detect a 2-fold or 0.5-fold difference of Rb as well as Cyclin-CDK (Cyclin D-CDK4/6), which is associated with G1 phase arrest (Supplementary Material) might explain the contradiction.

The reverse experiment using Trp53−/− MCs confirmed the target role of p53. Meanwhile, co-immunoprecipitation showed a interaction between HIF-1α and p53. Molecular docking showed that Lys328, Pro332, Arg245 and Lys251 of HIF-1α formed hydrogen bonds with Ala222, Tyr226, Glu225 and Asp265 of p53, respectively. The docking score was −28.063 kcal/mol, which was far lower than the assessment standard of −5 kcal/mol (Saikia and Bordoloi, 2019). These four sites may be the targets of the interaction between the two proteins. Madan et al. reflected that HIF-transcribed p53 chaperones HIF-1α in different cell lines of cancer under chronic hypoxia environment and demonstrated a novel pathway, where HIF-1α transcriptionally upregulates p53 by binding to five response elements in p53 promoter (Madan et al., 2019). This is consistent with our findings.

Finally, the *in vivo* experiments showed that roxadustat inhibited the proliferation of MCs via the HIF-1α/p53/p21 pathway *in vivo*, as well as improved the renal function of db/db mice. Whether increased HIF-1α is beneficial for the kidney? There are conflicting findings. Wang et al. showed that excessive activation of HIF-1α induced by chronic ischemia can mediate CKD (Wang et al., 2014). Stable expression of HIF-1α in mice by knocking out the *VHL* gene led to worse renal interstitial fibrosis in the 5/6 nephrectomy model (Kimura et al., 2008). Baumann et al. reported that knocking out the HIF-1α gene in podocytes could delay sclerosis of the glomerular (Baumann et al., 2016). However, in some studies, elevated HIF-1α showed benefits, and pretreatment of roxadustat reduced renal fibrosis through the Akt/GSK-3β/Nrf2 pathway (Li et al., 2020). Our finding is consistent with some of the reports, but contradicts the conclusions of others. Unlike previous studies that used high glucose, renal ischemia-reperfusion injury, and 5/6 nephrectomy models, increasing the level of HIF-1α actively rather than passively, like our study for a relatively long time, has shown positive effects on inhibiting proliferation and matrix deposition in the mesangial region.

**FIGURE 7:**
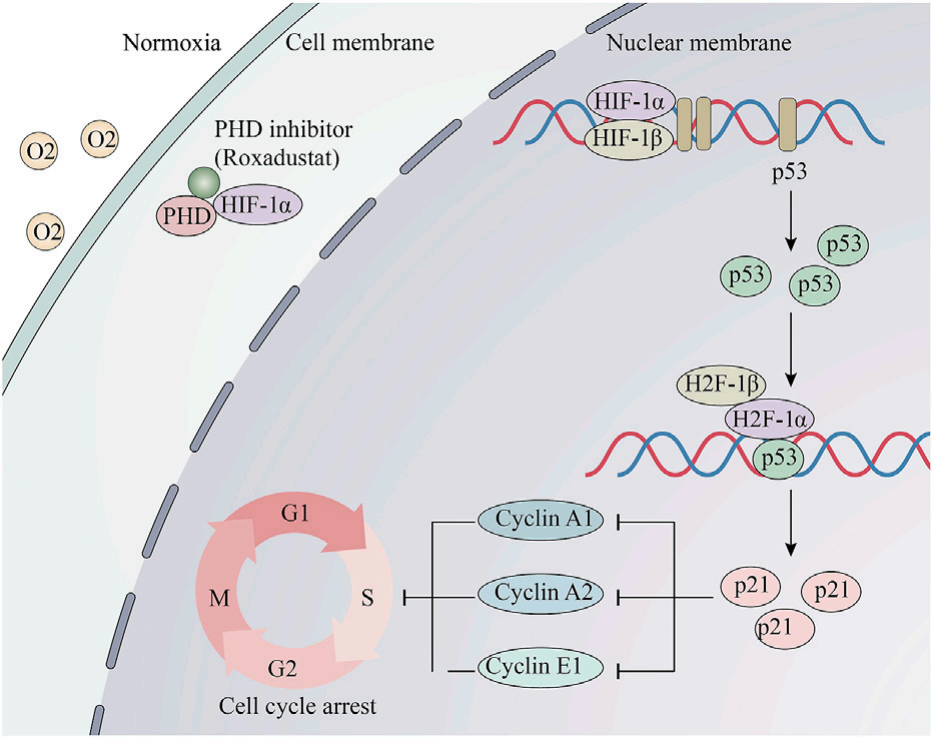
Schematic and potential mechanisms of Roxadustat’s anti-proliferative effect. Roxadustat stabilizes the protein level of HIF-1α under normaxia, then HIF-1α and HIF-1βbinds to HRE. Its downstream p53 gene is transcribed and translated. Then, HIF-1 combined with p53 binds to elements in the p21/CDNKN1A promoter and activates its transcription. Changes of the cell cycle proteins induced S-phase arrest.

In conclusion, when roxadustat was applied to increase the level of HIF-1α in MCs actively, continuously, and stably, some genes (e.g., p53), which cannot be activated in a short period, are activated. This may be the main reason for the observed effect of roxadustat (Figure 7). Besides, this approach might have an important translational value in clinical settings of early-stage DKD patients. Roxadustat might alleviate the symptoms of DKD by reducing mesangial cell proliferation via HIF-1α/p53/p21 pathway. However, there are some limitations: 1) we only used a single animal model (db/db mice); 2) the lifespan of db/db mice is much shorter than that of human, so the treatment duration was relatively short to mimic human DKD; 3) the safety of roxadustat in the early stage of DKD has not been proven, the potential side effects was not apparent. These limitations require further study and clinical verification.

## Data Availability

The original contributions presented in the study are included in the article/Supplementary Material, further inquiries can be directed to the corresponding authors.
