# Supplementary figures and images for: Roxadustat regulates the cell cycle and inhibits proliferation of mesangial cells via the hypoxia-inducible factor-1α/P53/P21 pathway
Source: Front Cell Dev Biol. 2025 Feb 18;13:1503477. doi: 10.3389/fcell.2025.1503477 (PMC11876171; doi:10.3389/fcell.2025.1503477)

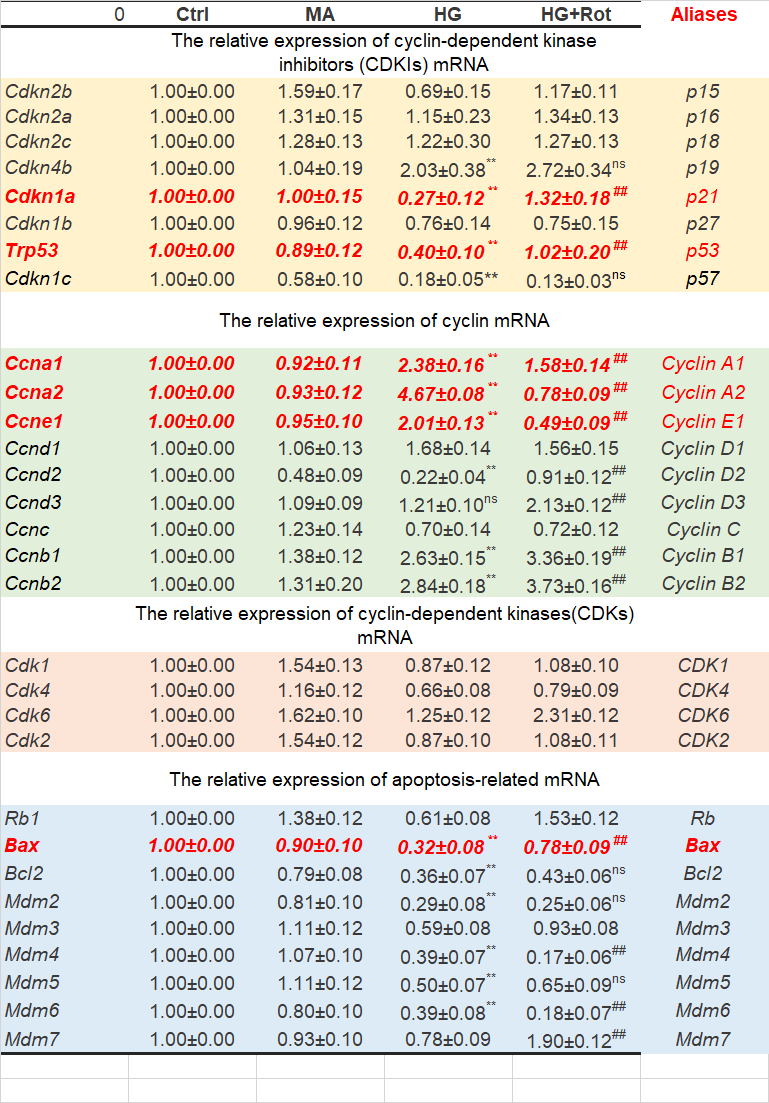

Supplement: Supplementary file 1 [file Image1.tiff]
